# Long term evolutions of hard exudates after anti-VEGF therapy for diabetic macular oedema

**DOI:** 10.1038/s41433-026-04408-1

**Published:** 2026-03-28

**Authors:** Kangyeun Pak, Changki Yoon, Srinivas R. Sadda

**Affiliations:** 1 Doheny Eye Institute, Pasadena, CA USA; 2 Department of Ophthalmology, Inje University Haeundae Paik Hospital, Pusan, Korea; 3 Department of Ophthalmology, David Geffen School of Medicine at UCLA, Los Angeles, CA USA; 4 Department of Ophthalmology, Seoul National University Hospital, Seoul, Korea

**Keywords:** Retinal diseases, Diabetes complications

## Abstract

**Purpose:**

To evaluate longitudinal change in hard exudate (HEs) volume over 5-years following anti-VEGF treatment for diabetic macular oedema (DMO).

**Methods:**

This study was a post-hoc analysis of structural optical coherence tomography (OCT) volume scans collected in the Diabetic Retinopathy Clinical Research Network Protocol T extension trial. A deep learning model was used to segment HEs and compute a HEs volume (mm³) for the entire OCT scan at baseline, 12, 24, 52, 104, 260 weeks (w). HEs volume was also quantified within the central subfield (CSF), inner ring (IR), and outer ring (OR) of ETDRS grid. Change in HEs over time was compared among treatment arms and a multiple regression analysis was used to evaluate the impact of HEs on visual outcomes relative to other biomarkers.

**Results:**

116 eyes were included (aflibercept: 44, bevacizumab: 30, ranibizumab: 42) in the final analysis. Across all studied regions, HEs significantly decreased through w52 (*p* < 0.001), and from w52 to w104, a further significant decrease was observed in the total macula and IR, though no additional reduction between w104 and w260. Change in VA at w260 was associated with baseline VA and w260 change in CST, but not with baseline HEs. At w52, HEs reduction was significantly greater with aflibercept and ranibizumab compared to bevacizumab, but no longer significant at w104 and w260.

**Conclusions:**

Following anti-VEGF therapy, HEs decreased through w104, but stabilised after that, and initial differences among agents disappeared by two years. The change in HEs did not predict w260 visual outcomes.

## Introduction

Diabetic macular oedema (DMO), affecting approximately 3–9% of diabetic patients, is a sight-threatening complication of diabetic retinopathy [1, 2]. Hard exudates (HEs), are frequently associated with DMO and manifest as yellow-white deposits in the retina resulting from a breakdown of the blood-retinal barrier [3]. The presence of HEs serves as a clinical marker for macular oedema and can contribute to photoreceptor and neuronal degeneration in the outer plexiform layer [4]. Consequently, monitoring HEs and their progression remains a subject of clinical significance.

Anti-vascular endothelial growth factor (anti-VEGF) therapy has become the standard treatment for DMO, demonstrating superior visual outcomes compared to laser photocoagulation and steroid therapy [5–7]. In addition, anti-VEGF therapy can improve macula anatomy by not only reducing retinal oedema, but also by restoring the positioning and integrity of the outer retinal bands (i.e. the photoreceptors), and by resolving HEs [8–12]. Recently, we reported the efficacy of anti-VEGF therapy in resolving HEs over a 1-year period and demonstrated a difference among agents [13, 14], but to our knowledge, the long-term impact of anti-VEGF therapy on HEs resolution has not been reported. Given the chronic nature of DMO, the persistence of HEs over time and their relevance of visual outcomes warrants study.

The Diabetic Retinopathy Clinical Research Network (DRCR.net) Protocol T extension study provides a valuable dataset for investigating this question[15]. The original DRCR.net protocol T trial [16] compared the efficacy of three anti-VEGF agents—ranibizumab, aflibercept, and bevacizumab—for the treatment of DMO over 2 years. In the Extension study, participants were asked to return at 5 years from randomisation to assess clinical outcomes. From years 2 to 5 after the randomised portion of the trial, the treatment protocol was not strictly controlled, and thus crossover to other anti-VEGF agents may have been performed at the investigators’ discretion. The results of Protocol T have profoundly influenced clinical practice. In addition, the imaging and other associated data collected in this trial have proven to be valuable for a number of significant post-hoc analyses [17, 18], though the long-term impact of therapy on HEs has not yet been evaluated quantitatively.

Thus, in this post-hoc analysis of the DRCR protocol T extension study, we aim to quantitatively assess the change in HEs volume over 5-years of anti-VEGF treatment for DMO and correlate the extent of HEs with visual outcomes.

## Methods

### Study design and participants

This retrospective post-hoc analysis was approved by the Institutional Review Board at the University of California, Los Angeles, and was conducted in accordance with the Health Insurance Portability and Accountability Act and the tenets of the Declaration of Helsinki.

Publicly-available deidentified data from the DRCR.net Protocol T Extension trial provided the cohort for this analysis [15]. To be included in this analysis, subjects had to have Cirrus structural optical coherence tomography (OCT, Carl Zeiss Meditec, Dublin, CA) volumes scans with OCTs available at baseline and at 12, 24, 52, 104, and 260 weeks(w) post-treatment, with no more than one missing visit among the follow-up time points. Exclusion criteria included: (1) a signal strength ≤ 3 (the minimum level required for the automated HEs segmentation algorithm); (2) poor image quality, for reasons other than low signal strength (e.g. poor centration, extension of retina beyond the vertical boundaries of the scan); (3) any deformation of the retinal pigment epithelium (RPE) contour aside from small drusen (of note, we have observed that a smooth RPE contour is important for accurate automated segmentation); (4) no HEs at baseline; and (5) presence or development of other retinal disease that could impact visual acuity (e.g. significant vitreo-retinal interface disease).

### HEs quantification and imaging analysis

HEs volume was measured using a previously reported method [19]. A deep learning model based on the U-Net architecture, trained on over 1811 manually annotated OCT B-scans, was used to segment HEs in each OCT B-scan. For quantification, HEs were defined as hyperreflective foci (HRF) consisting of three or more contiguous hyperreflective pixels located between the internal limiting membrane and the retinal pigment epithelium. The number of automatically segmented pixels was summed across 128 B-scans, yielding a volumetric representation in voxels. Based on the known scan dimensions (6 ×6 x 2 mm), the number of HEs voxels was converted to mm³. In addition to computation of the HEs volume within the entire scan, a projected en face map of segmented HEs was generated, and the ETDRS grid was overlaid, allowing quantification of HEs within the central subfield (CSF), inner ring (IR), and outer ring (OR).

### Data collection

The primary outcome measurement was the volume of HEs at baseline, 12, 24, 52, 104, w260 after treatment within total macula and each ETDRS region (CSF, IR, OR). Demographic and other clinical data were obtained from the DRCR.net database. Baseline variables that were considered included intervention arm, VA letter score, central subfield macular thickness (CST), age, sex, type of DM, mean arterial blood pressure, haemoglobin A_1c_ level, lens status, DM duration, body mass index, and prior laser history. CST was computed using the automated instrument software. The VA letter score was assessed at baseline and w260. CST values were assessed at each study timepoint. Changes in HEs, VA, and CST over time were also evaluated.

### Statistical analysis

Statistical analysis was performed using SPSS (version 12 for Windows; SPSS Inc., Chicago, IL, USA). For baseline HEs in the total macula, outlier values were truncated to three standard deviations (SDs) from the mean [20]. Intervention arm comparisons were performed using one-way ANOVA for continuous variables and chi-square tests for categorical variables. Turkey’s HSD test was used for post-hoc analysis of ANOVA. Paired t-tests were performed to compare HEs volume between each studied time point for the entire cohort and for each intervention arm. Linear regression analysis identified factors influencing visual acuity (VA) and change in VA, with variables meeting a univariate *p*-value threshold of ≤0.10 included in the multivariable model. Variables for univariate analysis included baseline VA, CST at each studied point, change in CST from baseline to each studied point, HEs volume at each studied point in each studied region, change in HEs volume from baseline to each studied point in each studied region, and demographic data. Stepwise regression was used and the model was adjusted so as not to infringe on multicollinearity. Data are presented as mean ± SD. For all tests, a value of *P* < 0.05 was considered statistically significant.

## Results

### Patient enrolment and baseline characteristics

Among 317 eyes originally enrolled in the Protocol T Extension trial, 120 met our inclusion criteria (Fig. S1). Four additional cases were excluded due to extreme baseline HEs values (defined as mean ± 3 SDs), resulting in a final cohort of 116 eyes (36.6% of original cohort). Among these, 44 received aflibercept, 30 received bevacizumab, and 42 received ranibizumab. The mean ages of patients treated with aflibercept, bevacizumab, and ranibizumab were 60.1 ± 10.0, 63.2 ± 8.2, and 59.0 ± 8.7 years (*p* = 0.154, respectively). Baseline total HEs of eyes treated with aflibercept, bevacizumab, and ranibizumab were 0.0284 ± 0.0328, 0.0212 ± 0.0251, and 0.0260 ± 0.0267 mm^3^, respectively (*p* = 0.568; ANOVA). Baseline characteristics of each treatment arm were well balanced except for prior history of anti-VEGF treatment, which was significantly higher in the bevacizumab arm (Table S1).

### Longitudinal changes in HEs

**Fig. 1:**
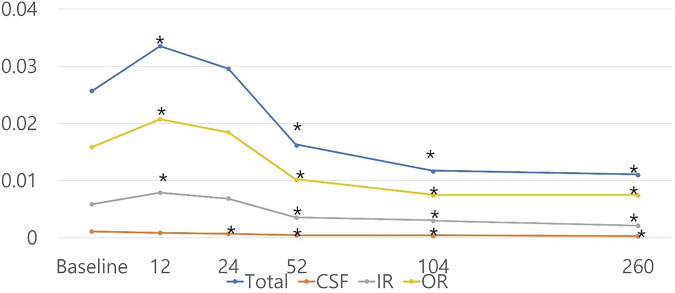
Overall Longitudinal Changes of Hard exudates in Total Cohort from Baseline to w260. Hard exudates demonstrated an initial increase in all studied regions but showed a significant decrease at w52 in all studied regions, with further reduction to w104 and persistent reduction without an increase through w260. Total: total macula; CSF central subfield, IR inner ring, OR outer ring. Asterisk indicates a statistically significant difference compared to baseline.

**Fig. 2:**
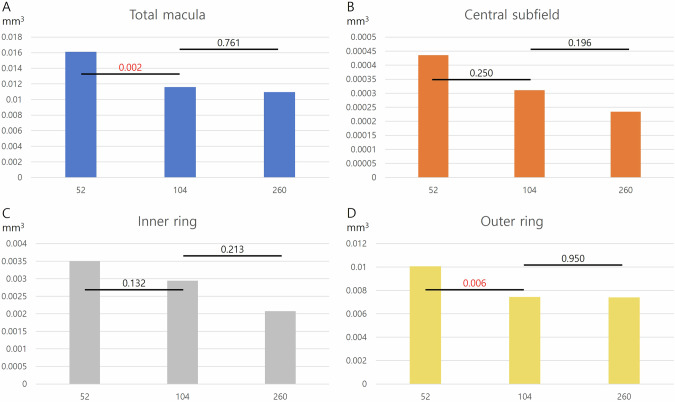
Change in hard exudate volumes over time. **A** In the total macula, hard exudates showed a continued significant decrease from w52 to w104, but no further reduction observed from w104 to w260. **B** In the central subfield, while numerically lower, no significant change in hard exudates was observed between w52 and w104, or between w104 and w260. **C** In the inner ring, despite a numerical reduction, there was no significant change in hard exudates from w52 to w104 and from w104 to w260. **D** In the outer ring, hard exudates continued to significantly decrease from w52 to w104 with no further decrease from w104 to w260. Bar graphs show HEs volumes at each time point. *P* values as shown are from pairwise comparisons between the bars as indicated by the line below each *P* value.

HEs demonstrated a statistically significant reduction at w52 across all studied regions compared to baseline (total macula: 0.0257 ± 0.0287 to 0.0161 ± 0.0263 mm^3^; CSF: 0.0011 ± 0.0033 to 0.0004 ± 0.0013 mm^3^; IR: 0.0059 ± 0.0070 to 0.0035 ± 0.0080 mm^3^; OR: 0.0158 ± 0.0196 to 0.0101 to 0.0160 mm^3^; *p* < 0.001 in all regions). No subsequent increase was observed during the five-year follow-up (Fig. 1). From w52 to w104, HEs continued to decrease, particularly in the total macula and IR (*p* = 0.002, 0.006, respectively). From w104 to w260, the observed reductions in HEs were not statistically significant in all regions (*p* = 0.196 – 0.950; Fig. 2). Complete numerical data and *p*-values are shown in Table 1.

**Table 1:** Longitudinal changes in hard exudates for the entire analysis cohort.

	Baseline	w12	w24	w52	w104	w260
Total macula	0.0257 ± 0.0287	0.0335 ± 0.0388	0.0296 ± 0.0395	0.0161 ± 0.0263	0.0116 ± 0.0212	0.0109 ± 0.0173
*p*-value		<0.001	0.168	<0.001	<0.001	<0.001
Central subfield	0.0011 ± 0.0033	0.0008 ± 0.0024	0.0007 ± 0.0020	0.0004 ± 0.0013	0.0003 ± 0.0006	0.0002 ± 0.0005
*p*-value		0.140	0.049	0.002	0.008	0.007
Inner ring	0.0060 ± 0.0071	0.0079 ± 0.0105	0.0068 ± 0.0105	0.0035 ± 0.0080	0.0029 ± 0.0072	0.0021 ± 0.0038
*p*-value		0.004	0.265	0.002	<0.001	<0.001
Outer ring	0.0159 ± 0.0196	0.0207 ± 0.0247	0.0184 ± 0.0256	0.0101 ± 0.0160	0.0074 ± 0.0126	0.0074 ± 0.0127
*p*-value		<0.001	0.147	<0.001	<0.001	<0.001

* *p*-values are compared to baseline.

### Factors Influencing VA and VA changes at w260

Multivariable regression analysis identified better baseline VA (adjusted beta = 0.411, *p* = < 0.001, partial R^2^ = 0.162), lower central subfield thickness (CST) at w260 (adjusted beta = -0.290, *p* = 0.001, partial R^2^ = 0.056), and shorter diabetes duration (adjusted beta = -0.219, *p* = 0.013, partial R^2^ = 0.045, adjusted R^2^ = 0.222) as independent predictors of favourable VA outcomes at w260. A lower baseline VA (adjusted beta = -0.331, *p* = 0.001, partial R^2^ = 0.077) and a smaller CST change from baseline to w260 (adjusted beta = -0.322, *p* = 0.001, partial R^2^ = 0.063, adjusted R^2^ = 0.276) were associated with greater VA gain at w260 (Table 2).

**Table 2:** Factors affecting visual acuity and change of visual acuity at 260 weeks.

Factors affecting visual acuity (Adjusted R^2^ = 0.222)			
Variables	Adjusted beta	*P* value	Partial R^2^
Baseline VA	0.411	<0.001	0.162
CST at w260	-0.290	0.001	0.056
Diabetes duration	-0.219	0.013	0.045

Factors affecting change of visual acuity (Adjusted R^2^ = 0.276)			
Baseline VA	-0.331	0.001	0.077
Change in CST at w260	-0.322	0.001	0.063

^*^Only valuables with *P* < 0.10 went on to the multivariable analysis.

*VA* Early treatment diabetic retinopathy study visual acuity, *CST* central subfield macular thickness.

### Comparative efficacy of three anti-VEGF agents

**Fig. 3:**
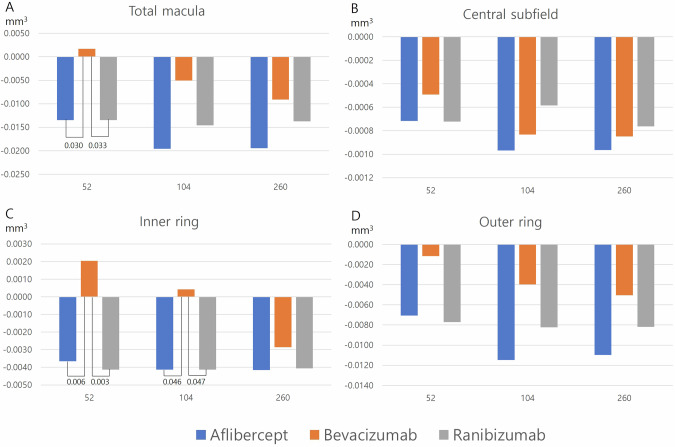
Comparative Efficacy of Three Anti-VEGF Agents. **A** In the total macula, both aflibercept and ranibizumab showed greater reduction in hard exudates than bevacizumab at 52w but the difference diminished after w104. **B** In the central subfield, there was no significant difference in the efficacy among agents. **C** In the inner ring, both aflibercept and ranibizumab showed greater reduction than bevacizumab at w52 and w104, but this difference did not persist through w260 **D** In the outer ring, there was no significant difference among agents.

In the total macula, the change in HEs over time was significantly different among agents (*p* = 0.019; ANOVA), with a greater reduction of HEs in eyes treated with aflibercept and ranibizumab compared to bevacizumab (*p* = 0.030 and 0.033, respectively) at w52. The difference was not significant at w104 and w260 (*p* = 0.077, 0.248, respectively, Fig. 3A). In the IR, the change in HEs over time among agents was significantly different at w52 and w104 (*p* = 0.002, 0.029, respectively; ANOVA) with a greater reduction for aflibercept (*p* = 0.006, 0.046, respectively) and ranibizumab (*p* = 0.003, 0.047, respectively) compared to bevacizumab. At w260, no significant differences remained among the treatment arms (Fig. 3C). In the CSF and OR, change in HEs showed no difference at w52, w104 and w260 among the three intervention arms (*p* = 0.209–0.963; Fig. 3B, D).

With aflibercept, HEs demonstrated a statistically significant reduction at w52 across all studied regions compared to baseline (*p* = <0.001–0.026). From w52 to w104, significant reduction was observed in the total macula and IR (*p* = 0.015, 0.016, respectively) From w104 to w260, the observed reductions in HEs were not statistically significant in all studied regions (Fig. S2).

With bevacizumab, the decrease in HEs was not significant at w52 across all studied regions compared to baseline (*p* = 0.268–0.749). From w52 to w104, significant reduction was observed in the total macula, IR, and OR (*p* = 0.010, 0.039, 0.007, respectively). From w104 to w260, the observed reductions in HEs were not statistically significant in any of the studied regions (Fig. S3).

With ranibizumab, HEs demonstrated a statistically significant reduction at w52 across all studied regions compared to baseline (*p* = 0.001–0.028). From w52 to w104, and w104 to w260, the observed reductions in HEs were not statistically significant in every studied region (Fig. S4). Complete numerical data and *p*-values are shown in Table S2.

## Discussion

In this post-hoc analysis of the DRCR.net Protocol T Extension study, we quantitatively assessed the longitudinal changes of HEs following anti-VEGF treatment for DMO over a five-year period. Our findings demonstrated a significant reduction of HEs at w52 across all studied regions, with a continued decline up to w104. Notably, no significant resurgence of HEs was observed throughout follow-up period, suggesting a sustained therapeutic effect of anti-VEGF agents on HEs. HEs or change in HEs, however, did not prove to be independent factors affecting visual outcomes in the multivariable analysis. There was a significantly different efficacy in the resolution of HEs among aflibercept, bevacizumab, and ranibizumab at 1 year. However, the difference diminished after 2 years.

The early resolution of HEs after anti-VEGF therapy aligns with previous reports, which indicated their efficacy in reducing macular oedema and associated pathological changes, including HEs. Domalpally et al. [12] reported a significant reduction of HEs using ranibizumab in a post-hoc study of the RISE and RIDE trials. Over a 24-month follow-up, monthly intravitreal ranibizumab resulted in significantly greater reduction of HEs area compared with sham. In the post-hoc analysis of BEVODEX study, Mehta et al. [21] compared the efficacy of bevacizumab and dexamethasone (DEX) implant on HEs over 24 months and concluded there was more rapid resolution of HEs with the DEX implant. We have also previously reported a decrease in HEs following anti-VEGF therapy over 1 year [9]. However, the long-term course of HEs following treatment has largely remained underexplored. Thus, our present results provide additional evidence supporting the role of anti-VEGF in achieving a sustained resolution of HEs over 5 years.

It is interesting that continued reduction of HEs was observed from w52 to w104 in the total macula and IR. However, the reduction plateaued from w104 to w260, with no statistically significant difference after w104. The decrease in HEs did lag behind the resolution of oedema, which is consistent with prior reports that indicating that HEs decrease gradually while CST decrease rapidly [12]. Unlike the rapid resolution of the fluid component of macular oedema, removal of lipid from the retina is thought to require the activity of phagocytic inflammatory cells [22]. It is noteworthy that no additional decrease was observed after 2 years even though more than half of HEs still remain at this timepoint (for example in the total macula, HEs decreased from a volume of 0.0257mm^3^ at baseline to 0.0161mm^3^ at w104). On the surface, this suggests that complete resolution of HEs is difficult with current therapies. One must note, however, that the treatment strategy was not rigorously controlled after the year 2 of Protocol T, and investigators could treat patients with the anti-VEGF agent of their choice and according to their own protocol. Thus, it is still possible that more aggressive treatments have yielded more complete HEs resolution. The functional consequence of this level of residual HEs is also uncertain.

Among the three anti-VEGF agents studied, aflibercept and ranibizumab demonstrated superior efficacy in reducing HEs compared to bevacizumab at w52, particularly in the total macula and IR regions. These findings are consistent with prior studies suggesting that aflibercept and ranibizumab may have more potent effects compared to bevacizumab [16]. However, by w104 and w260, inter-group differences in HEs reduction were no longer significant except for within the IR at w104. This suggests that while initial responses to anti-VEGF therapy may vary, long-term stabilisation of HEs occurs regardless of the specific agent used. Importantly, this loss of difference in HEs resolution among agents was achieved by w104, before lack of adherence to a strict protocol may have confounded comparisons among treatment arms. The underlying pharmacokinetic and pharmacodynamic differences among the three agents may contribute to this observation, with aflibercept and ranibizumab providing a more robust early response, while bevacizumab achieves comparable outcomes later. Interestingly, while bevacizumab did not result in significant reductions in HEs at w52, a delayed but significant reduction was observed between w52 and w104 catching up with aflibercept and ranibizumab by 2 year. These “catch-up” results are similar with CST and VA outcomes from DRCR.net protocol T study. This phenomenon is not surprising, considering retinal microvascular hyperpermeability as the main mechanism in the evolution of both macular oedema and HEs.

Multivariable regression analysis identified several independent predictors of visual acuity (VA) at w260, including better baseline VA, lower CST at w260, and shorter diabetes duration. These findings emphasise the importance of early diagnosis as patients with better initial VA and shorter disease duration demonstrated more favourable long-term outcomes. Additionally, the association between lower CST at w260 and better VA outcomes highlights the importance of continued treatment to resolve macular oedema. For VA changes at w260, lower baseline VA and smaller CST changes were associated with greater VA gains. This suggests that patients with poorer initial vision derive greater benefit from treatment, although their final VA may still be lower than that of patients with better baseline VA.

In the present analysis, HEs at baseline and change in HEs over time were not independent predictors for visual outcomes at w260. This stands in contrast to our previous report demonstrating that baseline HEs were an independent predictor for visual outcomes at 1 year (Pak KY, Yoon CK, and Sadda SR. (2025). Hard Exudates in Diabetic Macular Edema after Intravitreal Anti-VEGF Therapy: A Post-Hoc Analysis of the DRCR Protocol T Trial. Manuscript under revision). The reason why this relationship is lost over time is uncertain. We speculate that this may be related to the further resolution of HEs between year 1 and 2, which may impact the change in vision over time.

It should be noted, however, that there have been inconsistent results in the literature, regarding the relevance of HEs to visual outcomes. Chew et al. [4] reported that presence of HEs was associated with unfavourable visual outcome when the effect of DMO was adjusted. Domalpally et al. [12] reported that there was no correlation between VA and presence of HEs. On the other hand, the presence of HEs was independent factor for better VA in DRCR.net protocol I trial, and it was suggested that HEs may be indicative of a non-ischemic process [23]. We postulate that inconsistent methodologies (e.g. quantitative vs. qualitative, fundus photo vs. OCT) may explain these discrepancies.

We postulated several potential explanations for the lack of correlation between HEs and long-term visual outcomes in the present analysis. First, all therapeutics for DMO are also associated with a reduction in the retinal thickness, and this reduction in CST is a strongly correlated covariate, which makes it difficult to tease out an independent effect of HEs. In addition, HEs are mainly located in the parafoveal or extrafoveal regions (4.3% in the CSF, 23.0% in IR, and 61.5% in the OR with a baseline volume of 0.0257, 0.0011, 0.0059, 0.0158 mm^3^ in total macula, CSF, IR, and OR, respectively). Given that visual acuity will primarily depend on the status of the CSF, the direct impact of HEs may be difficult to identify given the small amount of CSF HEs in this cohort.

In the present analysis, we employed a previously validated deep learning algorithm for automatic identification and quantification of HEs from all B-scans in the volume. There have been previous efforts to quantify HEs using OCT. Some researchers, quantify HEs using an en face approach by measuring hyperreflective lesions within a midretinal slab(s) – this approach may underestimate the HEs extent, as multiple lesions within the same A-scan will only be counted once [11]. Manual segmentation of HEs on all B-scans within an OCT volume has also been reported but is tedious and time-intensive and thus not practical for large-scale studies [24]. Our deep learning approach has proven to be robust with a reported Dice coefficient of 0.727 and accuracy of 99.96%. We should note that other groups have also developed deep learning algorithms for HEs quantification [25, 26], and these automated approaches are now generally preferred over previous manual methods for HEs assessment.

Our study does have several limitations which should be acknowledged. First, this is a post-hoc analysis and the original study was neither designed nor powered to investigate questions concerning HEs. Second, our study is susceptible to selection bias as we only included approximately 40% of original protocol T extension cohort. Third, we were not able to definitely differentiate HEs from other potentially bright structures. The different origins of HRF include extravasated lipids (HEs) [27], microglia activated by inflammation [28], and migrating RPE cells [29]; though admittedly, migrated RPE cells are less likely to be present in eyes with DMO [30, 31]. Finally, as noted above, the treatment protocol was not tightly controlled between years 2–5, and change in therapy to alternative anti-VEGF agents and varying treatment regimens may confound comparisons after Year 2. On the other hand, most of the retinal oedema and HEs had resolved by Year 2, and thus further differences at Year 5 are probably unlikely even if a strict protocol was maintained. In spite of these limitations, our study has several strengths, including the use of a validated deep learning-based quantification method for HEs analysis, a long follow-up period, and a well-characterised cohort from a rigorously conducted clinical trial.

In conclusion, our study provides robust evidence for the sustained reduction of HEs following anti-VEGF therapy for DMO over 5 years. The initial superior efficacy of aflibercept and ranibizumab compared to bevacizumab in the resolution of HEs that we had previously reported, appears to diminish over time as a result of the protracted efficacy of bevacizumab. In the current study, neither the extent of baseline HEs nor the change in HEs over time was meaningfully correlated with long-term visual outcomes.

## Summary

### What was known before

- An early decrease in hard exudates associated with diabetic macular oedema after anti-VEGF treatment has been previously reported.
- Aflibercept and ranibizumab were shown to be superior to bevacizumab in resolving hard exudates at 1 year.
- Studies have reported inconsistent results regarding the correlation between baseline hard exudates and visual acuity.

### What this study adds

- This study adds important long-term data. Hard exudates significantly decreased for up to 2 years after anti-VEGF treatment, without a significant resurgence observed over a total 5-year follow-up period.
- The initial superior efficacy of aflibercept and ranibizumab over bevacizumab in resolving hard exudates diminished by the 2-year due to protracted efficacy of bevacizumab.
- Neither the baseline extent of hard exudates nor changes in hard exudates over time were meaningfully correlated with long-term visual outcomes, in contrast to findings through the first year.

## Supplementary information

Supplementary material

## Data Availability

The datasets generated during and/or analysed during the current study are available from the corresponding author on reasonable request.
