# Supplementary material for: Long term evolutions of hard exudates after anti-VEGF therapy for diabetic macular oedema
Source: Eye (Lond). 2026 Mar 28;40(9):1364–70. doi: 10.1038/s41433-026-04408-1 (PMC13269688; doi:10.1038/s41433-026-04408-1)
Supplement: Supplementary file 1 — Supplementary material [file 41433_2026_4408_MOESM1_ESM.docx]

Table S1, Baseline characteristics of the entire cohort and within aflibercept, bevacizumab, and ranibizumab groups.

| Variables | Total cohort | aflibercept | bevacizumab | ranibizumab | P value |
| --- | --- | --- | --- | --- | --- |
| Total number of eyes | 116 | 44 | 30 | 42 |  |
| Age, years | 60.5 ± 9.2 | 60.1 ± 10.0 | 63.2 ± 8.2 | 59.0 ± 8.7 | 0.154 |
| Sex (% of female), % | 48.3 | 40.9 | 53.3 | 54.8 | 0.340 |
| VA letter score | 67.1 ± 10.8 | 68.6 ± 9.4 | 65.0 ± 12.7 | 67.0 ± 10.6 | 0.384 |
| Macular thickness, Um | 445.2 ± 114.6 | 443.3 ± 111.0 | 445.7 ± 122.0 | 447.0 ± 115.7 | 0.989 |
| Intraocular pressure, mmHg | 15.4 ± 2.7 | 15.3 ± 2.4 | 15.3 ± 2.8 | 15.6 ± 2.9 | 0.868 |
| Mean arterial blood pressure, mmHg | 101.9 ± 12.6 | 102.1 ± 13.9 | 102.5 ± 12.5 | 101.3 ±11.3 | 0.923 |
| HbA1c | 8.05 ± 1.54 | 8.26 ± 1.48 | 7.74 ± 1.42 | 8.05 ± 1.68 | 0.370 |
| Lens status (% of phakic), % | 81.0 | 77.3 | 80 | 76.2 | 0.960 |
| Duration of diabetes, years | 16.7 | 15.8 | 18.3 | 16.5 | 0.568 |
| Prior anti VEGF treatment (% of yes), % | 10.3 | 4.5 | 23.3 | 7.1 | 0.033 |
| Body mass index, kg/m² | 33.60 ± 8.05 | 32.61 ± 7.41 | 34.38 ± 9.06 | 34.12 ± 8.05 | 0.631 |
| Prior PRP (% of yes), % | 11.2 | 9.1 | 13.3 | 11.9 | 0.633 |
| Prior focal laser (% of yes), % | 32.8 | 36.4 | 40 | 31.0 | 0.709 |

* Values were presented as mean±standard deviation

* p-values are between aflibercept, bevacizumab, and ranibizumab.

VA: Early treatment diabetic retinopathy study visual acuity; VEGF: vascular endothelial growth factor; PRP: Pan-retinal photocoagulation

Table S2. Longitudinal changes in hard exudates for aflibercept, bevacizumab, and ranibizumab groups.

|  | Baseline | w12 | w24 | w52 | w104 | w260 |
| --- | --- | --- | --- | --- | --- | --- |
| aflibercept | | | | | | |
| Total macula | 0.0284 ±  0.0328 | 0.0371 ±  0.0477 | 0.0338 ±  0.0514 | 0.0149 ±  0.0200 | 0.0086 ±  0.0088 | 0.0089 ±  0.0140 |
| p-value |  | 0.044 | 0.395 | 0.001 | <0.001 | <0.001 |
| Central subfield | 0.0012 ±  0.0034 | 0.0012 ±  0.0035 | 0.0009 ±  0.0028 | 0.0005 ±  0.0014 | 0.0002 ±  0.0004 | 0.0002 ±  0.0004 |
| p-value |  | 0.982 | 0.155 | 0.026 | 0.071 | 0.071 |
| Inner ring | 0.0060 ±  0.0073 | 0.0085 ±  0.0134 | 0.0064 ±  0.0101 | 0.0023 ±  0.0030 | 0.0019 ±  0.0027 | 0.0018 ±  0.0035 |
| p-value |  | 0.069 | 0.781 | <0.001 | 0.001 | <0.001 |
| Outer ring | 0.0173 ±  0.0228 | 0.0222 ±  0.0285 | 0.0218 ±  0.0343 | 0.0102 ±  0.0149 | 0.0057 ±  0.0066 | 0.0063 ±  0.0101 |
| p-value |  | 0.043 | 0.269 | 0.013 | <0.001 | 0.001 |
| bevacizumab | | | | | | |
| Total macula | 0.0212 ±  0.0251 | 0.0280 ±  0.0288 | 0.0304 ±  0.0352 | 0.0228 ±  0.0403 | 0.0139 ±  0.0351 | 0.0097 ±  0.0191 |
| p-value |  | 0.013 | 0.022 | 0.749 | 0.162 | 0.009 |
| Central subfield | 0.0012 ±  0.0046 | 0.0005 ±  0.0012 | 0.0007 ±  0.0018 | 0.0007 ±  0.0018 | 0.0002 ±  0.0006 | 0.0002 ±  0.0003 |
| p-value |  | 0.295 | 0.497 | 0.349 | 0.226 | 0.227 |
| Inner ring | 0.0045 ±  0.0062 | 0.0071 ±  0.0088 | 0.0086 ±  0.0146 | 0.0066 ±  0.0143 | 0.0045 ±  0.0130 | 0.0010 ±  0.0025 |
| p-value |  | 0.026 | 0.046 | 0.268 | 0.969 | 0.001 |
| Outer ring | 0.0136 ±  0.0185 | 0.0174 ±  0.0202 | 0.0175 ±  0.0203 | 0.0124 ±  0.0215 | 0.0076 ±  0.0188 | 0.0064 ±  0.0143 |
| p-value |  | 0.025 | 0.156 | 0.742 | 0.082 | 0.033 |
| Ranibizumab | | | | | | |
| Total macula | 0.0260 ±  0.0267 | 0.0336 ±  0.0343 | 0.0249 ±  0.0273 | 0.0125 ±  0.0178 | 0.0104 ±  0.0172 | 0.0093 ±  0.0160 |
| p-value |  | 0.017 | 0.685 | 0.001 | <0.001 | <0.001 |
| Central subfield | 0.0009 ±  0.0020 | 0.0007 ±  0.0014 | 0.0005 ±  0.0009 | 0.0002 ±  0.0004 | 0.0003 ±  0.0006 | 0.0002 ±  0.0003 |
| p-value |  | 0.200 | 0.091 | 0.028 | 0.029 | 0.018 |
| Inner ring | 0.0067 ±  0.0073 | 0.0078 ±  0.0076 | 0.0061 ±  0.0072 | 0.0025 ±  0.0041 | 0.0022 ±  0.0040 | 0.0023 ±  0.0046 |
| p-value |  | 0.279 | 0.597 | 0.001 | 0.001 | 0.001 |
| Outer ring | 0.0159 ±  0.0169 | 0.0215 ±  0.0234 | 0.0157 ±  0.0177 | 0.0082 ±  0.0122 | 0.0067 ±  0.0117 | 0.0058 ±  0.0111 |
| p-value |  | 0.008 | 0.919 | 0.001 | <0.001 | <0.001 |

* p-values are compared to baseline.Figure S1. Flow chart that summarizes the reasons for exclusions from the analysis


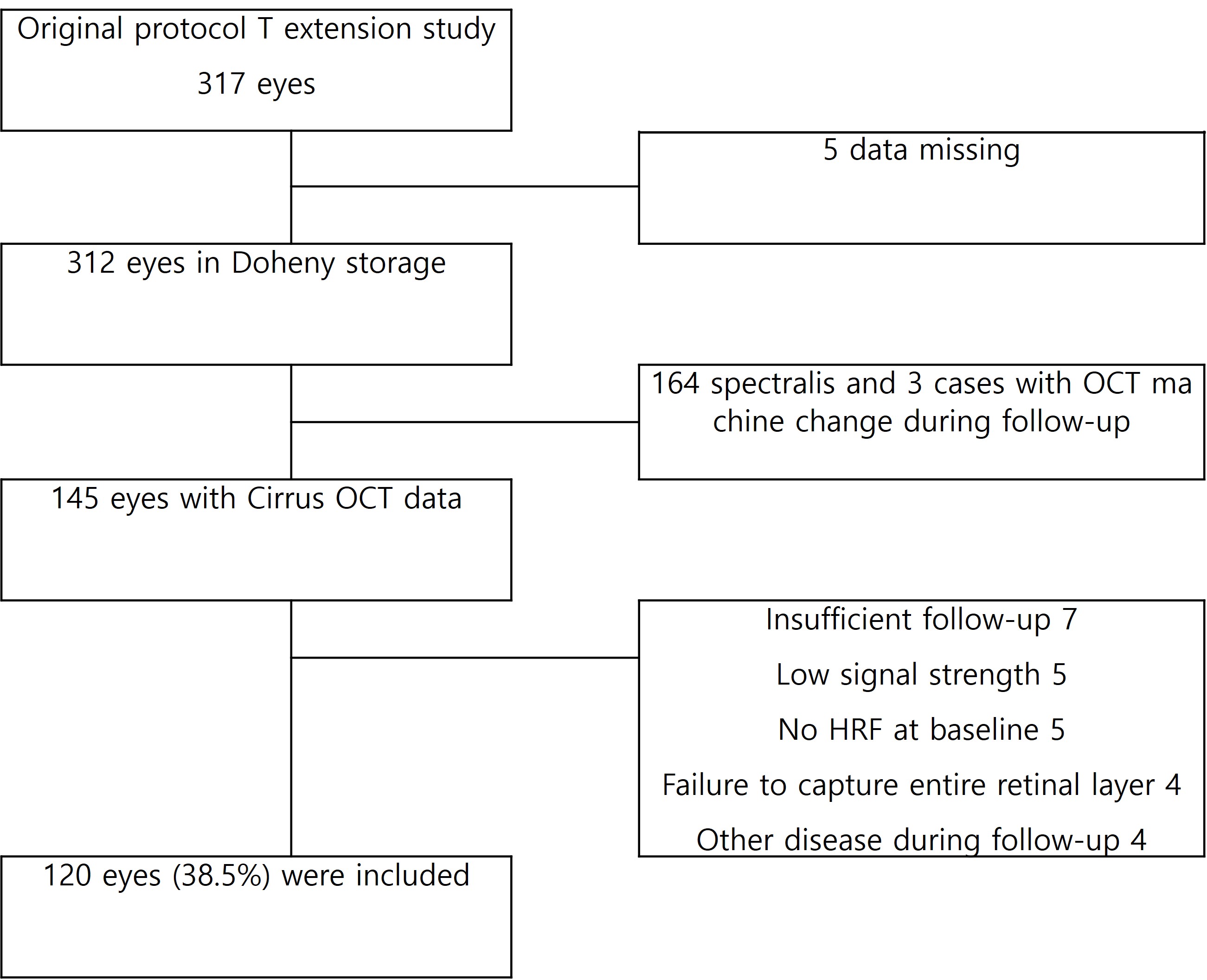


317 eyes were originally included in the protocol T extension study. Among them, data from 5 eyes was missing and thus 312 eyes were accessible. 167 eyes were excluded as the Spectralis OCT was used or there was a change in OCT device during the course of the study. Only Cirrus OCT scans were allowed in this analysis due to the requirement for dense OCT volumes. Among 145 eyes remaining eyes with Cirrus OCT data, 7 had insufficient follow-up, 5 had low signal strength, 5 eyes did not have HRF at baseline, in 4 eyes the full extent of the retina was not captured in the scan window, and 4 eyes developed other retinal disease during the follow-up --- these were excluded. Finally, 120 eyes (38.5%) met our inclusion criteria

Figure S2. Overall Time Course of Hard Exudate from Baseline to w260 According to Studied Region in Aflibercept


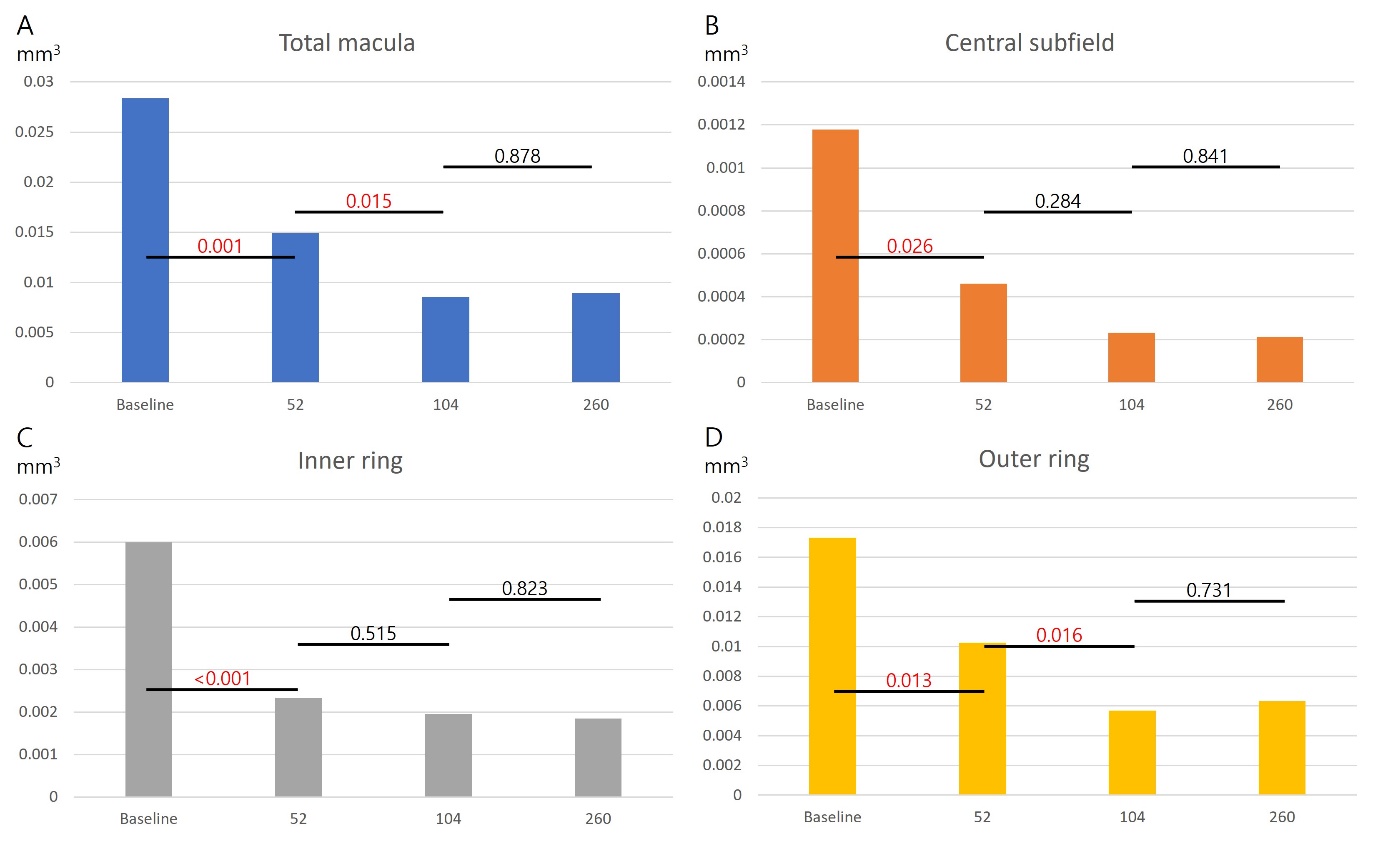


Bar graphs show HEs volumes at each time point. P-values as shown are from pairwise comparisons between the bars as indicated by the line below each P-value.

Figure S3. Overall Time Course of Hard Exudate from Baseline to w260 According to Studied Region in Bevacizumab


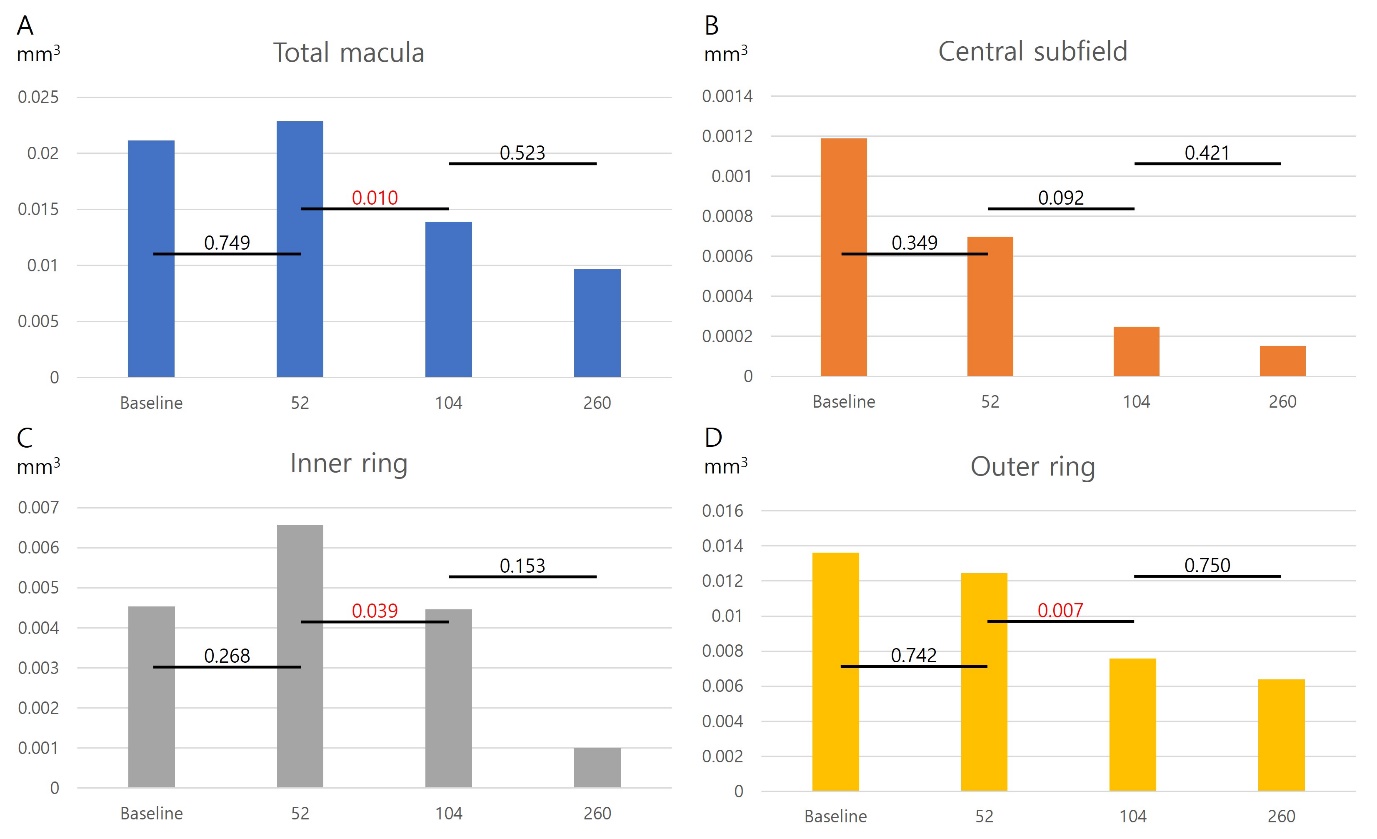


Bar graphs show HEs volumes at each time point. P-values as shown are from pairwise comparisons between the bars as indicated by the line below each P-value.

Figure S4. Overall Time Course of Hard Exudate from Baseline to w260 According to Studied Region in Ranibizumab


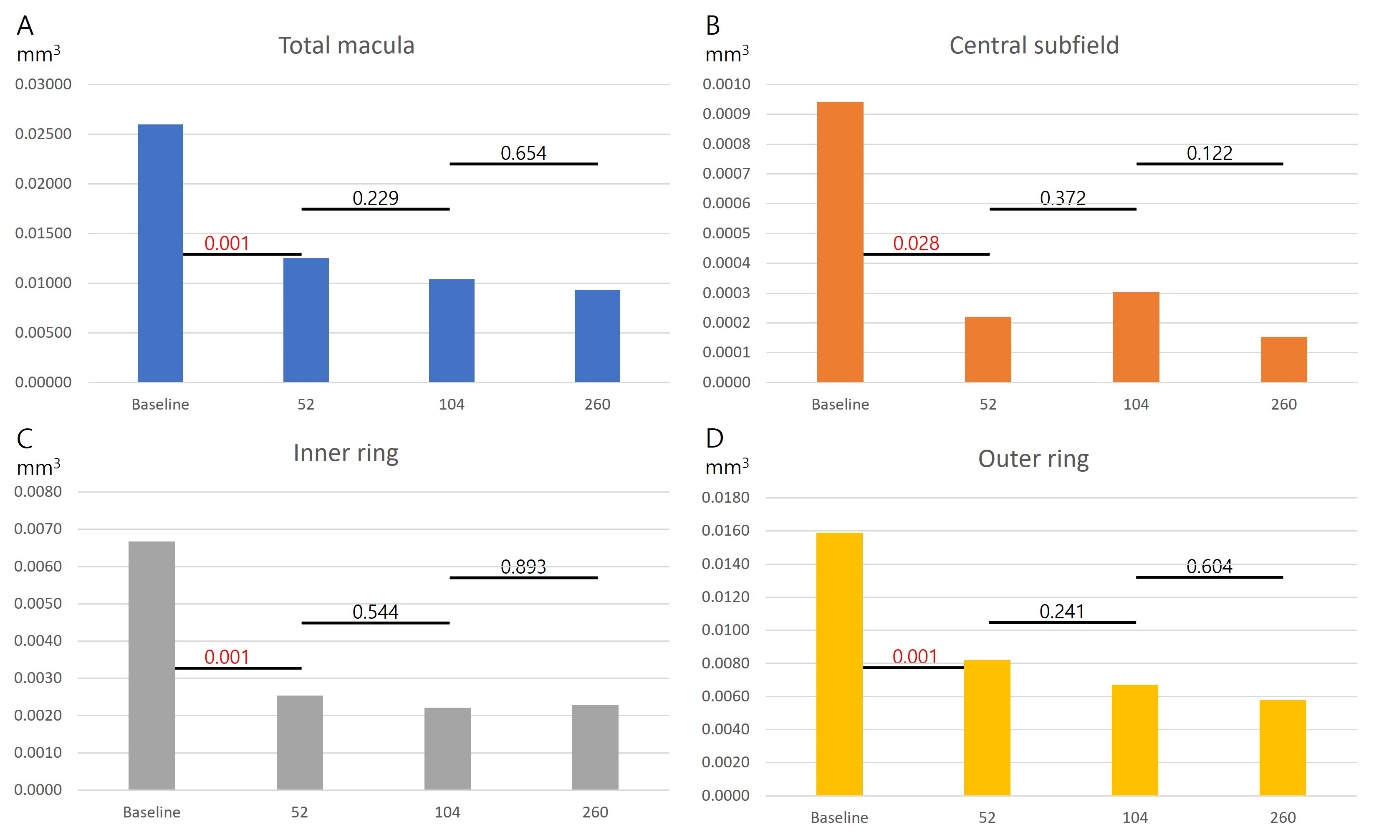


Bar graphs show HEs volumes at each time point. P-values as shown are from pairwise comparisons between the bars as indicated by the line below each P-value.
